# Health Behaviors and their Relationship with Disease Control in People Attending Genetic Clinics with a Family History of Breast or Colorectal Cancer

**DOI:** 10.1007/s10897-016-9977-2

**Published:** 2016-06-17

**Authors:** Annie S. Anderson, Stephen Caswell, Maureen Macleod, Robert JC Steele, Jonathan Berg, Jacqueline Dunlop, Martine Stead, Douglas Eadie, Ronan E. O’Carroll

**Affiliations:** 1 Centre for Research into Cancer Prevention and Screening, Division of Cancer Research, University of Dundee, Mailbox 7, Level 7, Ninewells Medical School, Dundee, DD1 9SY UK; 2 East of Scotland Genetics Service, Ninewells Hospital & Medical School, Dundee, DD1 9SY UK; 3 Institute for Social Marketing, University of Stirling, Stirling, FK9 4LA UK; 4 Division of Psychology, School of Natural Sciences, University of Stirling, Stirling, FK9 4LA UK

**Keywords:** Genetic, Cancer, Diet, Activity, Overweight, Fatalism, Control, Lifestyle

## Abstract

The current work aimed to assess health behaviors, perceived risk and control over breast/colorectal cancer risk and views on lifestyle advice amongst attendees at cancer family history clinics. Participants attending the East of Scotland Genetics Service were invited to complete a questionnaire (demographic data, weight and height, health behaviors and psycho-social measures of risk and perceived control) and to participate in an in-depth interview. The questionnaire was completed by 237 (49 %) of attendees, ranging from 18 to 77 years (mean age 46 (±10) years). Reported smoking rates (11 %) were modest, most (54 %) had a BMI > 25 kg/m^2^, 55 % had low levels of physical activity, 58 % reported inappropriate alcohol intakes and 90 % had fiber intakes indicative of a low plant diet. Regression analysis indicated that belief in health professional control was associated with higher, and belief in fatalism with poorer health behavior. Qualitative findings highlighted doubts about the link between lifestyle and cancer, and few were familiar with the current evidence. Whilst lifestyle advice was considered interesting in general there was little appetite for non-tailored guidance. In conclusion, current health behaviors are incongruent with cancer risk reduction guidance amongst patients who have actively sought advice on disease risk. There are some indications that lifestyle advice would be welcomed but endorsement requires a sensitive and flexible approach, and the acceptability of lifestyle interventions remains to be explored.

## Introduction

For people who are at greater risk of cancer due to a family history of the disease (which may reflect shared genetic and behavioral profiles) it is important to follow recommendations for cancer screening and lifestyle. NHS genetics clinics in Scotland offer early detection and counselling for people with a family history of breast cancer (BC) and colorectal cancer (CRC) and other (OC) cancers (Scottish NHS Genetics services http://www.healthsciencescotland.com/194_Scottish+NHS+Genetics+Centres.html) but offer little, if any, guidance on lifestyle.

It is estimated that 38 % of BC in post-menopausal women could be prevented by increased physical activity and reductions in alcohol intake and body fatness (World Cancer Research Fund 2015 http://www.wcrf-uk.org/uk/preventing-cancer/cancer-preventability-statistics). Observational studies show that BC and CRC risk is lowered with intentional weight loss (Ahn et al. 2007, Harvie et al. 2005) and data from bariatric surgery (Byers and Sedjo 2011) show that large weight losses are associated with sizeable reductions in female cancers (42 % reduction in overall cancer risk). Gramling et al. (2010) reported from the Women’s Health Initiative study that participating in healthy behaviors (higher physical activity, low alcohol intake and appropriate body weight) was beneficial for risk reduction in postmenopausal women and the degree of this benefit **was the same** for women with or without a family history of BC. Recent work by Nomura et al. (2016) has also reported that adherence to cancer prevention guidance (WCRF/AICR) is associated with lower breast cancer risk regardless of non-modifiable (taller height, family history of breast cancer, greater number of years of potential fertility and nulliparity) risk factor status.

It is estimated that around 47 % of CRC could be prevented by increases in dietary fiber and physical activity and reductions in consumption of red and processed meat, alcohol and body fatness (World Cancer Research Fund 2015 http://www.wcrf-uk.org/uk/preventing-cancer/cancer-preventability-statistics). For CRC, people with a family history may be more susceptible to lifestyle related risk. In a pooled analysis Cho et al. (2012) reported a greater association between CRC and alcohol consumption of ≥30 g/d among those with a family history of CRC (Relative risk (RR) 2.02 (95 % Confidence Interval (CI) 1.30, 3.13) compared to RR 1.23 (95 % CI 0.96, 1.57) for those with no family history). In a recent paper by Movahedi et al. (2015) in patients with Lynch Syndrome the association between obesity and CRC was significantly greater than that for underweight and normal-weight participants [RR 2.41(95 % CI, 1.22 to 4.85)], and CRC risk increased by 7 % for each 1-kg/m^2^ increase in body mass index.

In a UK survey of relatives of patients with CRC, Akhtar et al. (2008) reported that most (88 %) said they were prepared to make lifestyle changes if given enough information. In our BeWEL study (lifestyle intervention for people at higher CRC risk due to detection of an adenoma), 49 % of 997 patients actively requested further information about the lifestyle (weight loss) trial (Anderson et al. 2014).

It is recognized that informing people that they are at risk of developing a lifestyle related disease is rarely sufficient to change behavior (Wardle et al. 2000) and it is unlikely that knowledge of family history per se is sufficient to initiate weight management. Bostean et al. (2013) reported that whilst a family history of CRC and BC was associated with higher probability of cancer screening (especially for CRC), it was largely unrelated to health-related behaviors. In the few cases in which there was a significant association with lifestyle (e.g. physical activity in white males) people with a family history had **lower** odds of adherence to healthy lifestyle recommendations than those with no family history. In patients who go on to develop cancer, post operative complications associated with smoking have been well described but is also noteworthy that obesity is associated with poorer prognosis, increases in disease recurrence and overall mortality (Meyerhardt et al. 2003; Chan et al. 2014). In addition, it has been noted in the breast cancer care setting that women with higher BMI receiving neoadjuvant therapy are less likely to comply with chemotherapy and receive a lower total dose which in turn is associated with a reduced likelihood of a complete pathological response (Fontanella et al. 2015).

In a review of behavioral responses to genetic information on risk, Marteau and Lerman (2001)) argues that motivation to change behavior may be achieved by increasing beliefs that changing behavior **can** reduce risks and that the individual believes they have the ability to change. Risk perceptions can be subdivided into absolute likelihood, comparative risk and anticipated conditional risk (Cameron et al. 2012). There is also compelling evidence that health locus of control beliefs are a powerful determinant of health behavior e.g. the belief that responsibility lies with the individual (internal), powerful others (e.g. health professionals) or that health outcomes are largely determined by chance/fatalism. In a study of 7000 young adults from 18 countries, the odds of engaging in healthy behavior were more than 40 % greater among individuals in the highest vs. lowest quartile of internal locus of control, and high chance/fatalism locus scores were associated with more than 20 % reductions in the likelihood of healthy behaviors (Steptoe and Wardle 2001). Current evidence from effective behavior change programs highlights the importance of self-efficacy and self-regulatory techniques (e.g. goal setting) to promote change (Dombrowski et al. 2012; Michie et al. 2013).

Health behaviors are clearly related to morbidity and mortality. Khaw et al. (2008) assessed over 20,000 adults using a simple baseline assessment of 4 health behaviors. At 11 year follow-up, the mortality risk for those with four compared to zero health behaviors was equivalent to being 14 years younger in chronological age.

The National Institute for Clinical and Health Excellence (NICE) guidelines on familial BC recommend that a lifestyle information leaflet is provided (NICE 2013), but this is unlikely to be sufficient to assist weight loss per se. A recent Cochrane review has highlighted that communicating DNA based risk estimates does not lead to changes in health behavior (Hollands et al. 2016). In Scotland, genetic counsellors are not trained to promote health protective behaviors, nor is there a definitive guideline on lifestyle advice. This results in a discussion of lifestyle only if requested by patients and provision of advice that is ad hoc, and severely time restricted. A recent trial of a lifestyle intervention program with people at increased risk of colorectal cancer (due to a diagnosis of colorectal adenoma) has demonstrated that clinically relevant outcomes can be achieved through lifestyle change (Anderson et al. 2014). It is plausible that such programs may also be relevant in the family history clinic setting, although little is known about current health behaviors amongst attendees or their interests and beliefs about lifestyle and cancer prevention.

## Aim

To assess modifiable health behaviors associated with increased risk of breast/colorectal cancer and psycho-social perspectives which may impact on these behaviors in order to inform the design of a lifestyle intervention amongst attendees of family history clinics.

## Objectives

In a family history clinic setting:

1. To assess current smoking, diet, alcohol, activity and anthropometric measures associated with cancer risk and compare these to health guidelines.
2. To explore salient beliefs about perceived cancer risk, control of health and concepts of fatalism in relation to current health behaviors.
3. To evaluate comprehension and expectations about health behaviors, motivations, barriers to and facilitators of lifestyle change and views on possible lifestyle intervention programs.

## Methods

### Participants and Data Collection

Participants, over age 18, attending the East of Scotland Genetics Service (ESGS) family history clinics who had not had a cancer diagnosis were eligible for inclusion. Patients scheduled for screening appointments in the family history breast mammographic screening or colonoscopy screening clinics during the survey period were mailed a study pack invitation with their routine appointment letter. The invitations were sent to patients under regular review and new patients. Existing users of the genetic cancer screening service (who were not scheduled for appointments within the survey period) were also sent the mailed study pack with a covering letter from the ESGS family history clinic. Study packs comprised a participant information sheet (PIS), letter of endorsement from the ESGS family history clinic and an anonymized questionnaire on lifestyle to be returned to the research center by the enclosed stamped address envelope (SAE). In addition, an invitation to participate in an in-depth interview on lifestyle and cancer risk (with separate SAE envelope to maintain questionnaire anonymity) was included.

### Sample Size

Study size was pragmatically based on a review of users of the service which was reported as around 700 patient visits for cancer related family history assessments per year. We estimated that around 40 % of patients would participate (*n* = 280) and we aimed to carry out a total of 20 in-depth interviews. All people responding positively to the interview invitation were contacted in order to recruit participants within the time frame available.

### Questionnaire Measures

The self-completion questionnaire provided data on:

1. Demographics - gender, age, ethnicity, marital status and education. Post code was used to assess Scottish Index of Multiple Deprivation (SIMD) – a categorical system of identifying social position based on area of residence which takes account of housing, crime, access to services, education, health, income and employment (Ralston et al. 2014).
2. Self-reported height and body weight
3. Lifestyle measures pertinent to cancer prevention (not specific to CRC or BC) were assessed as markers of adherence to cancer prevention guidelines.

#### Smoking

Participants were asked to report smoking status (and number of cigarettes smoked by current smokers).

#### Alcohol Intake

was estimated using a 7 day recall questionnaire to indicate how many drinks (beer/wine/fortified wine/spirits) they had consumed over the previous seven days. This total was then recoded (Emslie et al. 2009) to provide an approximate number of units of alcohol.

#### Physical Activity

was estimated using the using the short form International Physical Activity Questionnaire (IPAQ). The IPAQ assesses walking, activities of moderate and vigorous intensity as estimates of frequency (days per week) and duration (time per day). These are combined (http://www.ipaq.ki.se/scoring.pdf) to provide a summation of duration (in minutes) and frequency (days) and for the purpose of this study participants were then categorized as “active”, meeting at least one of

- 3 days of 20 min vigorous activity/week
- 5 days of moderate/walking 30mins
- 5 or more days of any combination of walking, moderate or vigorous activity achieving a minimum of at least 600 MET minutes/week

or “inactive” if neither a, b or c were achieved across a seven day self-reported period.

#### Red and Processed Meat

(beef burgers, sausages, liver products, savory pies, corned beef, ham, luncheon meat and bacon) consumption was estimated by frequency of consumption scales using the relevant questions in the validated EPIC food frequency questionnaire (European Prospective Investigation of Cancer 2016, http://www.srl.cam.ac.uk/epic/nutmethod/FFQii.shtml) and average UK portion measures for adults (Wrieden and Barton 2006).

#### Plant Foods

In the absence of a total dietary assessment the validated DINE questionnaire (Roe et al. 1994) was used to estimate dietary fiber intake. This short questionnaire enables foods rich in dietary fiber to be assessed and a dietary fiber intake “score” to be calculated based on the fiber content of standard portion sizes, weighted by frequency of consumption. A fiber score of less than 30 (‘low’) is equivalent to a fiber intake of 20 g/day or less, whilst over 40 (‘high’) is equivalent to an intake of more than 30 g/day.

A health behavior score was then calculated where +1 was scored for each health measure which was in accordance with behavioral recommendations for cancer prevention by the World Cancer Research Fund (WCRF) (http://www.wcrf-uk.org/uk/preventing-cancer/cancer-preventability-statistics). The domains scored were smoking, body fatness, alcohol intake, physical activity, red and processed meat consumption and plant food proxy (dietary fiber) No weighting was applied to domains. The possible score ranged from 0 to 7 points (higher score = engaging in greater number of healthy behaviors).

Psycho-social measures explored two main domains, risk perceptions and health locus of control, using validated questionnaire assessments. Cancer risk was assessed with three separate constructs:

- “Absolute likelihood” (e.g. “How likely do you think it that, at some point in your life, you [will/would] get colon/breast cancer?”) assessed by two items with a total possible score of 0–12.
- “Comparative risk” (i.e. “Compared to the average person of your age and gender, what would [be] your risk of getting breast/colon cancer at some point in your life?”) assessed with this one item with a possible score of 0–6.
- “Anticipated conditional risk” (i.e. What do you think would be your chance of getting breast/colon cancer in your lifetime if you were to stick to a healthy lifestyle?) assessed with this one item with a possible score of 0–6 (Cameron et al. 2012).

Three further domains were assessed:

- Internal health locus of control (e.g. The main thing which affects my health is what I myself do)
- Perceived health professional control (e.g. Health professionals control my health)
- Perception of chance/fatalism (e.g. My good health is largely a matter of good fortune).

Each was assessed by three questions on a five point Likert scale (possible score 3–15 for each domain). [Helmer et al. 2012]

### Interview Measures

The in-depth interviews used a semi-structured interview discussion guide to explore perceptions and understanding of cancer risk, perceptions of the role of lifestyle and views on being offered lifestyle advice (Appendix 1). Interviews were carried out by two experienced researchers and digitally audio-recorded with the participants’ written consent.

### Data Analysis

#### Questionnaire

responses were analyzed using SPSS 21 (IBM Corp. Released 2012. IBM SPSS Statistics for Windows, Version 21.0. Armonk, NY: IBM Corp). Descriptive statistics were used for socio-demographic characteristics.

Analysis of the relationship between psychosocial constructs and the total number of lifestyle behaviors was assessed using linear regression. Pearson correlations were also carried out to test the relationship between risk and health locus of control measures.

#### Qualitative

In–depth interviews were transcribed and a thematic analysis was conducted. The approach drew on both the deductive and inductive approaches to thematic analysis. Themes relating to the pre-specified research questions (for example, attitudes towards receiving lifestyle advice) were actively sought in the data, whilst further themes evolved from the coding process itself.

Ethical approval was granted by the East of Scotland Research Ethics Committee (REF no 14/ES/1091).

## Results

In total, 484 study invitations were sent over the 7 month recruitment period and 237 (49 %) questionnaires were returned. Responses were fairly evenly distributed between participants recently invited to genetic screening (53 %) and those under longer term surveillance (47 %). The majority of respondents (70 %) had a family history of breast cancer, 26 % of colorectal cancer and 4 % had a history of multiple cancers.

Participants ranged in age from 18 to 77 (mean 46 ± 10 years) and 88 % were female. Most were white (99 %) and 78 % were educated to professional or university degree level. Two thirds (66 %) lived in the two least deprived quintiles of The Scottish Index of Multiple Deprivation (SIMD) (Scottish Government n.d.) (Table 1).

**Table 1:** Sociodemographic characteristics of participants

	Breast Cancer Screening *n* = 165	Colorectal Cancer Screening *n* = 61	All ^a^ *n* = 237
Age (years) Mean (SD)	45.4 (10.0)	46.5 (10.1)	45.8 (10.1)
Missing	4		5
Gender			
Male	1 (1)	27 (44)	28 (12)
Female	165 (99)	34 (56)	209 (88)
Ethnicity			
White	163 (98)	61 (100)	233 (99)
Non white	1 (1)	0 (0)	1 (0)
Missing	2 (1)	0 (0)	3 (1)
Marital Status			
Single	23 (14)	6 (10)	30 (13)
Married/co-habiting	125 (75)	49 (80)	181 (77)
Widowed/divorced/separated	16 (10)	6 (10)	24 (10)
Missing	2 (1)	0 (0)	2 (1)
Educational attainment			
Secondary school	35 (21)	16 (26)	52 (22)
Other professional/technical	56 (34)	24 (39)	86 (36)
University degree	73 (44)	21 (34)	97 (41)
Missing	2 (1)	0 (0)	2 (1)
Scottish Index of Multiple Deprivation Quintiles			
1 (most deprived)	12 (7)	4 (7)	17 (7)
2	12 (7)	6 (10)	20 (8)
3	31 (19)	9 (15)	41 (17)
4	61 (37)	23 (38)	87 (37)
5 (least deprived)	47 (28)	18 (30)	67 (29)
Missing	3 (2)	1 (2)	5 (2)

a Includes 10 people attending for a combination of cancers

All data are n (%) unless stated otherwise

### Assessment of Current Lifestyles

Most (89 %) participants reported being non-smokers and 84 % were consuming meat within the red meat limits. However, only 45 % achieved recommended guidance for physical activity, 45 % reported a BMI in the normal range 18.5 to 25 kg/m^2^ (23 % of the participants had a BMI > 30 kg/m^2^) and 42 % reported appropriate alcohol intake. Less than 10 % had estimated fiber intakes indicative of a high plant diet and 8 % reported refraining from processed meat. There were no differences in these health behaviors by breast or colorectal family history (Table 2). The mean health behavior score for all participants was 3.14 (SD 1.1, range 1 to 7).

**Table 2:** WCRF cancer prevention recommendations and participant achievement of these recommendations

Recommendation	Met recommendations in this study if:	Breast Cancer Screening *n* = 165		Colorectal Cancer Screening *n* = 61		All^a^ *n* = 237	
		n	Achieving n (%)	n	Achieving n (%)	n	Achieving n (%)
Alcohol: Limit alcohol drinks to one per day for women, two per day for men	≤1 drink/per day for women, ≤2 drinks/day for men	165	72 (44)	61	22 (36)	236	100 (42)
Body fatness: Be as lean as possible within the normal range of body weight	BMI ≥ 18.5 and ≤25.0	156	75 (48)	59	24 (41)	225	102 (45)
Fibre: Eat mostly foods of plant based origin	DINE fibre score > 40	138	12 (9)	55	7 (12)	222	19 (9)
Physical Activity: Be physically active	IPAQ ≥30 min moderate 5 days	156	72 (45)	60	24 (40)	231	103 (45)
Processed meat: Avoid	Avoid	165	15 (9)	59	2 (3)	236	18 (8)
Red meat: Limit intake	<500 g/week	158	134 (85)	60	47 (78)	228	191 (84)
Smoking: avoid	Non smoker	166	149 (90)	60	54 (90)	236	210 (89)
Mean score (0–7)		166	3.19 (±1.14)	61	2.95 (±1.0)	237	3.14 (±1.1)

a Includes 10 people attending for a combination of cancers

### Beliefs about Control over Disease Risk

Responses for absolute likelihood risk ranged from 0–6, comparative risk and anticipated conditional risk scores ranged from 0–6 (Table 3). Locus of control ranged from 4–15 and both clinician control and fatalism variables had responses in the range 3–15. No significant differences were detected between clinical groups. The three perceived risk scores were all highly inter-correlated (all *r* > 0.80) therefore a single mean perceived risk score was calculated.

**Table 3:** Psychosocial scores by cancer screening

Domain and constructs	Possible score	Breast Cancer Screening *n* = 165		Colorectal Cancer Screening *n* = 61		All^a^ *n* = 237	
		n	Mean (SD)	n	Mean (SD)	n	Mean (SD)
Cancer risk							
Absolute likelihood risk	0–6	160	3.8 (1.3)	57	3.9 (1.4)	226	3.8 (1.34)
Comparative risk	0–6	161	3.8 (1.4)	57	4.0 (1.3)	227	3.9 (1.4)
Anticipated conditional risk	0–6	160	3.5 (1.2)	57	3.5 (1.2)	226	3.5 (1.2)
Locus of control	3–15	160	11.1 (2.3)	60	10.8 (2.5)	230	11.0 (2.4)
Clinician control	3–15	158	5.3 (2.4)	60	5.7 (2.8)	228	5.5 (2.6)
Fatalism	3–15	160	6.9 (2.5)	59	7.7 (2.9)	228	7.1 (2.6)

a Includes 10 people attending for a combination of cancers

In order to test the predictive ability of the main psychological constructs (perceived risk and health locus of control) after controlling for demographic variables and type of clinic attended, we ran a linear multiple regression analysis, predicting the total health behavior score (range 0–7).

In the first model we entered the demographic variables of age, gender and SIMD. In the second model we added the type of clinic attended. In the third and final model we added locus of control (internal control, health professional control and chance/fatalism) and mean risk perception score.

The final regression model was not significant (F (8204) = 1.495, *p* = 0.161) and only explained 6 % of the variance in total health behaviors. The only predictors that were significant in the final model were health professional control (Beta = 1.971, *p* = 0.050) and chance/fatalism (Beta = −1.986, *p* = 0.048), indicating that the belief in health professional control was associated with a higher and chance/fatalism with a lower total health behavior score.

### Comprehension and Expectations about Health Behaviors and Risk

Face to face interviews were completed by 20 people. Participants were predominantly female (95 %), aged 32 to 70 years. Most (65 %) were recruited in relation to a history of breast cancer risk.

All interviewees appeared aware that they were at some level of elevated risk compared to the general population, although their understanding of their risk status (and their willingness to articulate and discuss it) appeared to vary considerably. Ways of dealing with this knowledge varied considerably, ranging from fatalism (“what will be will be”) and denial (“I KNOW I won’t get it”) through to proactive efforts to reduce risk in other ways; for example, one female participant had had elective breast surgery.

Only smoking and UV exposure were widely accepted as clear cut cancer risk factors. There were some doubts expressed as to the link between lifestyle and breast cancer, and when prompted few were familiar with the available evidence on controllable risk. Alcohol and diet in general terms were mentioned as possible risk factors by some; on further prompting as to the nature of the link with diet, some ventured that fat was a problem and should be cut down, red meat and processed meat were mentioned by the two patients with elevated colorectal cancer risk, and fruit and vegetables were also mentioned in connection with cancer in general. “Keeping yourself active” and physical activity tended to be mentioned only after considerable prompting, although those few participants who did mention activity seemed to believe that the link was credible.

Few reported having made any lifestyle changes specifically because of their diagnosis/elevated risk. The one male participant said that he had cut down on processed meat “because of nitrates”, prompted by a conversation with the colorectal consultant. A female participant who was carrying a BRCA2 gene mutation and had higher risk of ovarian and breast cancer put a high value on diet and exercise, both for herself and her family, although she implied that she did not expect that this could in any way cancel out or reduce her elevated genetic risk. It was more common to make lifestyle changes for other reasons, such as in relation to diabetes, or reaching a milestone age which prompted a re-examination of lifestyle in general.

Recall of having been offered lifestyle advice during the clinic visit and subsequent screening was generally limited. Many did not recall any mention of lifestyle, and one commented, that, on reflection, it was “odd” that clinic staff concerned with cancer risk and prevention did not discuss health behaviors.

A few inferred from the questions asked at the clinic, about smoking, alcohol and so on, that clinic staff assumed there was a link between lifestyle factors and risk, but it seemed that this was not made explicit. The few who did recall any lifestyle advice recalled it as an incidental part of the conversation with nurses and clinicians.

### Response to the Concept of Lifestyle Advice Being Offered

Although no participants expressed explicit opposition to or dislike of the concept of lifestyle advice being offered during the family history clinic process or subsequent screening, levels of enthusiasm and interest were very variable. There appeared to be a relationship between control beliefs and interest in lifestyle advice, with more fatalistic participants being generally less interested; one younger woman, for example, pulled a face in response to the suggestion that clinic staff could advise about diet, responding “life is for living”. In contrast, the one male participant, who was able to articulate his level of risk in scientific terms, expressed the view that it would make rational sense to attempt to reduce risk in those areas where it could potentially be controlled, such as certain aspects of lifestyle.

Some said that learning about how lifestyle factors might relate to their own specific cancer risk would make such advice more relevant and interesting than general health education advice which they had heard before and did not necessarily believe. Others said that they might be interested in lifestyle advice if it was linked to other ongoing health concerns such as weight gain and thyroid problems, but implied that they would be put off by any discussion linking lifestyle advice to their own cancer risk. A participant who had a BRCA2 gene mutation commented that “5 % here or there” (the implication being that this was the difference that lifestyle advice might make, at best) was of little relevance in her own situation. She acknowledged that lifestyle advice may be of interest and relevance to those with a different risk profile to herself, but it was clear that any such advice offered to her herself could have had the potential to add further distress. Of the lifestyle topics which could be discussed with patients in this setting, it appeared that there was most potential interest in dietary advice, as this could be beneficial in other areas such as weight management. It was clear that this was a sensitive area and that if lifestyle advice were to be given more systematically in future, in this setting, it would need to be given with regard to the receptivity, personality and beliefs of each individual patient.

## Discussion

It is likely that questionnaire respondents had a heightened awareness of cancer and risk, and therefore might be expected to be highly motivated to adopt health behaviors concordant with risk reduction. Our data suggest that health behaviors related to diet, activity and obesity were largely similar to the general Scottish population where decades of public health initiatives have failed to bring about major changes in lifestyle. However, the relatively low rates of smoking (11 %) reported in this study are encouraging and are lower than those found in the general population (21 %) (Scottish Health Survey 2014) and similar to those of people of higher socio-economic status. These findings may relate to wider awareness of the risks, severity of the impact of smoking on cancer risk, and the greater likelihood of clinicians discussing this behavior.

Other studies of the health behaviors of people participating in cancer genetics clinics or with a family history of cancer have similarly found that while some self-reported health behaviors are ‘better’ than in the general population, overall health behaviors remain a concern (Emmons et al. 2000; Lemon et al. 2004). One possible explanation is that this group, despite having heightened awareness of cancer and regular contact with cancer screening services, are no better informed than the general population about the relationship between lifestyle risk factors and cancer, or are unconvinced of the relationship. Our study found evidence to support this, in that qualitative interview participants had some uncertainty regarding the role and importance of lifestyle factors in relation to cancer, and recalled little discussion of lifestyle behaviors having taken place in their consultations with clinic staff.

Several other studies have similarly found limited awareness of lifestyle risk factors for cancer in populations with a family history of cancer (Begum et al. 2009, Spector et al. 2011), or have found that limited discussion of lifestyle factors occurs in cancer genetics and family history clinic settings with patients taking their own responsibility to seek out health information (Spector 2007). It is plausible that time pressures in clinical settings do not allow a full lifestyle assessment and advice to be provided and there may be few opportunities to refer to other health professionals (beyond smoking cessation services). We have reported elsewhere (in relation to patients with colorectal cancer) that clinicians lack confidence, skills, time and conviction (about health improvements) to discuss obesity related health behaviors (Anderson et al. 2013) and this may also be true within the family history setting.

Previous research has suggested that perceived risk, engaging in healthy behaviors and beliefs about control are connected. For example, McLeish et al. (2013) reported that women who had a close relative with a BRCA mutation (but did not themselves have the mutation), who perceived their own breast cancer risk to be high (> 50 %), reported significantly more changes in health behaviors following risk assessment than those with lower risk perceptions (irrespective of professionally estimated risk). In the current study we did not find a simple association between perceived risk and engaging in healthy behaviors although we do not have any measure of changes that may have occurred after risk assessment.

In the current study, the greater belief in health professional management of risk, the more likely it was for respondents to have healthier lifestyles. This observation highlights the potential importance of clinical endorsement for healthy ways of life. Conversely, and as expected, strong fatalistic views were associated with unhealthy behaviors and this is consistent with findings elsewhere. For example, O’Carroll et al. (2001) found that fatalistic beliefs were the best predictor of delayed presentation following myocardial infarction.

In the genetics clinics service, there is the opportunity to elicit and potentially modify beliefs about illness, risk and control. Such brief interventions in other clinical settings are beginning to show promise (Petrie et al. 2002; O’Carroll et al. 2013; O’Carroll 2014), and a trial of this approach based in the genetics clinic setting may be warranted. Previous work by McLeish et al. (2013) in women attending breast cancer family history clinics reported that most women were willing to adopt changes in lifestyle (e.g. more exercise, less alcohol), although the authors noted that younger women (<40 years) and those with daughters had made fewer spontaneous changes and that “special attention” would have to paid to this subgroup “if their good intentions are to be translated into action”.

The findings from the qualitative interview data suggest that, while cancer family history and genetics clinics offer a potential opportunity for lifestyle advice and support which is currently under-used by clinic staff, any advice would need to be offered with care and sensitivity. In our study, patients varied widely in their willingness to articulate and discuss risk and their feelings of control. The need for more education on lifestyle was discussed by women with a BRCA1 or BRCA2 mutation in a study by Spector (2007), but it was noted that this should be offered by healthcare providers as part of a health education plan offering tailored strategies and resources.

In conclusion, current health behaviors in this group of patients are incongruent with current cancer risk reductions guidance. There are some indications that patients would welcome lifestyle advice but endorsement requires a sensitive and flexible approach that takes account of patients’ state of mind and receptivity to discussing lifestyle-related risk, personally relevant triggers and motivations (particularly regarding weight loss), and current circumstances (e.g. advice on physical activity appropriate to ability and disability, childcare responsibilities, social support and cost appropriate opportunities for engaging in interventions).

The feasibility and acceptability of delivering lifestyle interventions remains to be explored and offers considerable scope for an active approach to cancer risk reduction.

### Study Limitations

The current work is an exploratory study from one site in Scotland and may not be representative of the country overall. However, we are unaware of any genetics clinics in Scotland where detailed lifestyle advice covering all modifiable factors associated with increased cancer risk is discussed. The questionnaire methodology relied on reported (in contrast to measured) health behaviors with considerable scope for reporting bias, which may mean that lifestyle could potentially be poorer than indicated. The questions used to assess psycho-social measures were limited in order to reduce participant burden and did not permit an in-depth analysis of risk perceptions per se and the final regression model predicting health behavior was not significant. The qualitative methodology was not designed to be representative but to identify and illustrate a range of issues relevant for approaching lifestyle guidance. It was notable that detailed probing about understanding of risk (both in general and at specific level) was limited by the discomfort exhibited by some participants during the interviews.

### Research Recommendations

The response of people attending family history clinics (due to concerns about breast and colorectal cancer) to personalized lifestyle advice is unknown. The current study provides formative perspectives on which to build an appropriate intervention program for feasibility testing. Future research in this area should be informed by randomized clinical trials of lifestyle intervention with objectively measured behavior change end points (e.g. body weight change) in the first instance, together with cost effectiveness analysis.

### Practice Implications

Even where time by counsellors is limited, all patients who are seeking advice on cancer risk are likely to gain from positive guidance on lifestyle. In the UK, health promotion within the National Health Service (NHS) has focused on concepts of “Making Every Contact Count” (https://www.gov.uk/government/uploads/system/uploads/attachment_data/file/216423/dh_132114.pdf) and “Every healthcare contact is a health improvement opportunity” (http://www.healthscotland.com/documents/4128.aspx). These initiatives encourage regular conversations in clinic based on behavior change, empowering healthier lifestyle choices and exploring the wider social determinants that influence all of our health. Such approaches deserve further exploration in the genetic clinic setting and might include brief interventions described as the “5 A’s” which has been widely used in smoking cessation. This approach involves *asking* approval to discuss relevant behaviours, *advising*, *assessing* willingness to change, *assisting* with facilitating the change (this might be a referral to community pharmacy or health professional) and *arranging* follow up contact (e.g letter, telephone) (Agency for Healthcare Research and Quality 2016). The use of brief interventions for smoking and excess alcohol consumption are already well described within primary care consultations (Stead et al. 2013, Kaner et al. 2007).

It is recognized that there is some general skepticism about the outcomes of health behavior interventions delivered by health professionals. However, it is notable that both overweight and obese people are more likely to report participating in weight management efforts when a health professional has given some advice on the topic (Jackson et al. 2013). There is now considerable evidence that points to the positive success of interventions that include evidence-based behavioral change techniques (e.g. goal setting, self- monitoring, social support both within (Anderson et al. 2014, Short et al. 2013) and out with (Avery et al. 2015) cancer settings.

A recent European study of general practitioners (Julian-Reynier et al. 2015) and breast surgeons on breast cancer risk communications has highlighted that lifestyle BC risk factors such as obesity and alcohol were rarely/occasionally mentioned, (although this differed by country and the specialty of the providers involved). The authors highlight that risk communication skills should be improved during the initial and vocational training of health care professionals and the findings of the current work support this recommendation.

## Conclusion

The results suggest that current health behaviors in this group of patients who have actively sought advice on disease risk could be improved. The feasibility and acceptability of delivering lifestyle interventions remains to be explored.

## Appendix 1

**Table:** Perceptions of cancer risk: interview discussion guide

1. Background Personal circumstances: age, place of residence, family circumstances, current health, any other medical conditions, occupation 2. Recall and experiences of attending the Genetic Risk Assessment Clinic (GRAC) and, if relevant, subsequent clinic for breast cancer or colorectal cancer Perceptions and understanding of why they were referred; trigger event or other (if self-referred) Motivation for attending the clinic/s; trigger event or other Initial expectations of what they would experience and learn at the clinic/s, including emotions, anxieties, hopes, fears, any specific questions. Recall of what actually happened and what was discussed Perceptions of and satisfaction with the overall experience in terms of: ● quality of interactions with staff, ● how any tests or investigations were conducted ● clarity of information given, ● understanding of advice given ● usefulness and relevance of advice Explore awareness and recall of having received letter and any leaflets 3. Perceptions of cancer risk Prior to attending the clinic/s, perceptions of the main risk factors for site-specific cancer: explore the perceived role/importance of: ● family history/biology, ● fate/chance, ● lifestyle factors ● other factors Perceptions and beliefs regarding whether and to what extent they feel cancer risk can be reduced, and if so how Explore understanding of risk information in general in the context of illness What levels of risk are considered high, moderate, acceptable? Explore whether some risk factors are perceived to have more weight or to be less susceptible to modification than others Perceptions of the trustworthiness and credibility of the risk information presented at the clinic/s Perceptions of their own risk of cancer before attending the clinic/s. Probe how they conceptualise this risk if at all, eg. in relation to general population Whether (and if so, how) this changed as a result of their clinic experience 4. Cancer risk and lifestyle (If not already spontaneously covered) Probe any awareness of lifestyle risk factors for cancer in general. If yes, how did they become aware of or first heard about the potential link between lifestyle and cancer (probe whether heard from friend/family, advised by health professional, etc) In turn, explore awareness of any specific advice/messages in relation to cancer prevention, believability of the advice, and perceptions of how easy or difficult it would be to act on. Explore awareness of and response to the concept that for a specific cancer, genetic factors may account for some proportion of an individual‟s risk and lifestyle factors for another proportion. Is this a graspable concept? Does it seem likely, believable? Following on from this, how much of the risk would need to be lifestyle related for people to feel it was worth acting on? What else if anything do they feel that people can do to reduce risk of cancer? 5. Response to the concept of lifestyle advice Have they ever sought or been offered lifestyle advice, for example on diet, weight, smoking, physical activity? In what context/with what motivation? When do they think getting advice about lifestyle might (or might not) be important, relevant, meaningful (in context of engagement with cancer family history clinics)? If they would welcome lifestyle advice, probe views on possible formats, eg. face-to-face session, leaflet, telephone call, group.	
